# Development of an Indirect ELISA for Serological Diagnosis of *Bovine herpesvirus 5*

**DOI:** 10.1371/journal.pone.0149134

**Published:** 2016-02-11

**Authors:** Luana A. Dummer, Itauá L. Araujo, Fabrício S. Campos, Matheus C. da Rosa, Paula F. Finger, Patricia D. de Oliveira, Fabricio R. Conceição, Geferson Fischer, Paulo M. Roehe, Fábio P. L. Leite

**Affiliations:** 1 Laboratório de Bacteriologia, Centro de Desenvolvimento Tecnológico - Núcleo de Biotecnologia, Universidade Federal de Pelotas, Pelotas, RS, Brazil; 2 Laboratório de Virologia, Universidade Federal do Rio Grande do Sul, Porto Alegre, RS, Brazil; 3 Laboratório de Imunologia Aplicada, Centro de Desenvolvimento Tecnológico - Núcleo de Biotecnologia, Universidade Federal de Pelotas, Pelotas, RS, Brazil; 4 Laboratório de Virologia e Imunologia Animal, Faculdade de Veterinária, Universidade Federal de Pelotas, Pelotas, RS, Brazil; 5 Fundação Estadual de Pesquisa Agropecuária, Saúde Animal - Instituto de Pesquisas Veterinárias Desidério Finamor (IPVDF), Eldorado do Sul, RS, Brazil; Rowan University, UNITED STATES

## Abstract

Bovine herpesviruses 1 and 5 (BoHV-1 and BoHV-5) are economically important pathogens, associated with a variety of clinical syndromes, including respiratory and genital disease, reproductive failure and meningoencephalitis. The standard serological assay to diagnose BoHV-1 and BoHV-5 infections is the virus neutralization test (VNT), a time consuming procedure that requires manipulation of infectious virus. In the present study a highly sensitive and specific single dilution indirect ELISA was developed using recombinant glycoprotein D from BoHV-5 as antigen (rgD5ELISA). Bovine serum samples (*n* = 450) were screened by VNT against BoHV-5a and by rgD5ELISA. Compared with the VNT, the rgD5ELISA demonstrated accuracy of 99.8%, with 100% sensitivity, 96.7% specificity and coefficient of agreement between the tests of 0.954. The rgD5ELISA described here shows excellent agreement with the VNT and is shown to be a simple, convenient, specific and highly sensitive virus-free assay for detection of serum antibodies to BoHV-5.

## Introduction

Bovine herpesviruses 1 and 5 (BoHV-1 and BoHV-5) are economically important pathogens associated to a variety of manifestations, including respiratory and genital disease, reproductive failure and meningoencephalitis. Presently, BoHV-1 is subdivided into three different genotypes: 1 (BoHV-1.1), 2a (BoHV-1.2a) and 2b (BoHV-1.2b), whereas BoHV-5 is subdivided in genotypes a (BoHV-5a), b (BoHV-5b) and c (BoHV-5c) [1–3]. Infection with such viruses occurs at the mucosal surfaces and after replication at infection sites, viruses penetrate local sensory nerves and gain access to ganglia. Both can establish latent infections, usually in sensory ganglia that innervate the primary site of infection [4,5]. However, BoHV-5 is apparently more neuropathogenic than BoHV-1, although the latter may eventually cause neurologic disease. When clinically apparent encephalitis/meningoencephalitis is manifested, the outcome is usually fatal, affecting cattle of different age groups, leading to important economic losses in South America [6,7]. In contrast, BoHV-1-associated respiratory disease is usually manifested with low mortality and high incidence of clinical disease [5,8,9]. Nevertheless, most bovine herpesvirus infections are subclinical [10].

Molecular and immunological studies indicate that the two virus types differ in their antigenic profile; although their protein repertoire averages 82% amino acid identity [11], there are genomic regions less conserved between these viruses, such as those presented at the carboxy-terminal regions of viral envelope glycoproteins C (gC) and D (gD) [12,13]. Phylogenetic comparisons between BoHV-1 gD (gD1) and BoHV-5 gD (gD5) demonstrate that, on average, their carboxy-terminal region is 64.1% identical [13], but among BoHV-5 gD, the identity level is 95.8% and, among BoHV-1 gD, 98.3%, indicating that this regions are highly conserved within each viral species, yet less conserved between BoHV-1 and BoHV-5 [13]. However, the differentiation of antibody responses induced by these two virus types by conventional serological techniques is not possible due to high degree of antibody cross-reactivity between the two viruses [2,3,14,15].

Serological detection of antibodies to BoHV-1 or BoHV-5 is commonly performed using the virus neutralization test (VNT) [16,17]. However, VNT cannot be automated and demands manipulation of infectious virus, requiring cell culture facilities and a great deal of labor, making it time-consuming (3–5 days) and expensive [18]. An alternative to VNT is the use of ELISAs for antibody detection, which are designed to be fast, sensitive and relatively inexpensive. In addition, ELISAs can easily be applied for large scale screening of test samples and allow the use of purified recombinant viral envelope proteins as antigenic substrate; which can be produced in large quantities without handling live viruses and can eliminate the presence of host cellular proteins in the test, reducing false-positive reactions (reviewed in reference [19]).

Viral envelope glycoproteins of BoHV-1 and BoHV-5, especially the glycoproteins B (gB), C (gC) and D (gD) interact with receptors of permissive cells in early stages of infection. The gD interaction with its receptor is required for viral envelope fusion with membranes of host cells and is essential for this virus infectivity (reviewed in reference [20]). Its role in the initial stages of BoHV-1 and BoHV-5 infection and its abundance in the viral envelope make gD a major target for the stimulation of antibodies by the host’s immune system [21], thus, gD seems a suitable candidate antigen for the development of immunodiagnostic assays.

In this study, recombinant BoHV-5 gD (rgD5) expressed in *Pichia pastoris* [22] was used as antigen in the development of an indirect ELISA (rgD5ELISA), which was further evaluated in its performance by comparison with conventional VNT results.

## Materials and Methods

### Expression and purification of the recombinant gD5

The cloning of the BoHV-5 (strain SV507/99) glycoprotein D into *P*. *pastoris* strain KM71H Mut^S^ expression system, as well as the expression of the recombinant glycoprotein D (rgD5) were previously described [22]. Briefly, the recombinant clone was inoculated in culture flasks containing buffered glycerol-complex (BMGY) medium (1% yeast extract, 2% peptone, 1.34% yeast nitrogen base, 0.00004% biotin, 1% glycerol, 100 mM potassium phosphate pH 6.0) and incubated in orbital shaker for 24 h at 28°C with agitation speed of 150 rpm. The cells were harvested and resuspended in 1/10 (10%) of the original culture volume of buffered methanol-complex (BMMY) medium (BMGY medium with 0.5% methanol in replacement of 1% glycerol). To induce expression, 1% of 100% methanol were added every 24 h, and cells were kept in the same growth conditions described above for 72 h. Cells were then harvested and the collected supernatant concentrated with Centriprep 50YM (Millipore) device at 1,500 x *g* for 10 min at 20°C. The rgD5 was then purified by affinity chromatography using both HisTrap HP 1 mL columns pre-packed with pre-charged Ni Sepharose and the ÄKTAprime Automated Liquid Chromatography system (GE Healthcare).

### SDS-PAGE and Western blot analysis

To monitor the purification process and confirm rgD5 antigenicity prior to the ELISA development, SDS-PAGE was carried out on 12% polyacrylamide gel. The gels were either stained overnight with Coomassie Blue R-250 (Bio-Rad) or electroblotted onto nitrocellulose membrane (Bio-Rad) using Bio-Rad Mini Trans-Blot Cell (Bio-Rad). Briefly, the membrane was blocked with 5% non-fat milk and, after three washes in phosphate buffer saline containing 0.05% Tween-20 (PBS-T) (137 mM NaCl, 2.7 mM KCl, 100 mM Na_2_HPO_4_, 2 mM KH_2_PO_4_, pH 7.4), the membrane was incubated with mouse monoclonal antibody (MAb) anti-6xHis HRP conjugated (Invitrogen) or with polyclonal antibody (PAb) anti-BoHV-5 from bovine experimentally immunized with inactivated BoHV-5 as previously described [22]. The immunoblot was developed using Sigma *FAST* 3,3’-Diaminobenzidine (DAB) with Metal Enhancer tablets (Sigma). The protein concentration was determined by bicinchoninic acid (BCA) protein assay (Pierce) method according with the manufacturer’s instructions.

### Cell and viruses

Madin Darby bovine kidney cells (MDBK, originally ATCC CCL22) were grown in Eagle’s Minimal Essential Medium (MEM; Life Technologies) supplemented with antibiotics (200 IU/mL streptomycin and penicillin, 5 μg/mL enrofloxacin and 2.5 μg/mL amphotericin B) and 10% fetal bovine serum (CultiLab) at 37°C in a 5% CO_2_ humidified atmosphere. Stocks of BoHV-5a strains RP and EVI 88/95 [23] were propagated in MDBK cells until cytopathic effect (CPE) was visible in 90% of the monolayers. Infected cells were then frozen, thawed, clarified at low speed centrifugation and supernatants stored at -80°C. Prior to virus neutralization test, the virus stocks were titrated and stored in liquid nitrogen.

### Development of the rgD5ELISA

In order to establish the optimal concentration of antigen and the optimal serum dilution to be tested, different concentrations of purified rgD5 as well as different control serum concentrations were screened in checkerboard titrations. The recombinant glycoprotein was diluted in 0.05 M carbonate-bicarbonate buffer (pH 9.6) to concentrations ranging from 200 to 12.5 ng/well of rgD5 and was tested against varying serum dilutions (1:100 to 1:500), prepared in PBS-T. Assay conditions were as follows: 96-well microtiter plates (Nunc-Immuno MicroWell PolySorp, Nunc) were coated overnight at 4°C with purified rgD5. The plates were washed three times and incubated for 1 h at 37°C with blocking solution (5% of non fat dry milk + 3% of casein in PBS-T). After three washes, serum samples diluted in PBS-T were added in duplicate to the wells; and the plates were then incubated for 1 h at 37°C. After three additional washes, horseradish peroxidase (HRP)-conjugated rabbit anti-bovine immunoglobulin G (IgG; Sigma-Aldrich) was diluted as appropriate (1:5,000 in PBS-T) and added to plates, followed by incubation at 37°C for 1.5 h. Subsequently, plates were washed five times with PBS-T and 100 μL/well of peroxidase substrate o-phenylenediamine dihydrochloride (OPD; Sigma-Aldrich) diluted in 0.07 mM citrate-phosphate buffer (pH 4.2) with 0.03% (v/v) of H_2_O_2_ was added. Plates were incubated 15 min in the dark and the reaction stopped with 2 N H_2_SO_4_. Results were read using ELISA TP-Reader plate spectrophotometer (ThermoPlate) at an optical density (O.D.) 492 nm.

### rgD5ELISA

Once the optimal conditions for the rgD5ELISA assay were established, 96-well plates were coated with 50 ng/well of purified rgD5 (100 μL volume) as described above. Bovine sera (*n =* 450) diluted 1:400 in PBS-T were individually placed in wells in duplicate (100 μL volume per well). Sera from cattle of Hereford, Red Angus or mixed Hereford breeds, with ages varying from two months to seven years, obtained between 2009 and 2013, were collected through jugular or caudal venipuncture and stored at -20°C prior to use in the diagnostic procedures. Samples were originated from farms located in the State of Rio Grande do Sul, southern Brazil (S1 Table). Positive and negative samples to BoHV-5a (as defined by VNT) were used as controls.

### Virus neutralization test (VNT)

Serum samples used in the ELISA development were tested for neutralizing antibodies to BoHV-5a as previously described elsewhere [10,24]. Briefly, after inactivation, sera were serially diluted in MEM (1:2 and 1:4) in 96-well plates (TPP) in duplicate. A BoHV-5a virus suspension containing 100 cell culture 50% infective doses (CCID_50_) in 50 μL volumes was added to each well and incubated at 37°C in a 5% CO_2_ humidified atmosphere. Subsequently, approximately 3×10^4^ MDBK cells/well were added and the plates incubated until 100 CCID_50_ were observed in the wells with the virus back titration. Results were expressed as either positive or negative to neutralizing antibodies against BoHV-5a, with basis on the detection/neutralization of virus induced CPE.

### Statistical analysis and graphs

Receiver-operating characteristics (ROC) curves, the area under the curve (AUC), the determination of optimal cut-off point, the calculation of the positive and negative predictive values (PPV and NPV, respectively) and the agreement test (inter-rater agreement—κ coefficient) were conducted with the software MedCalc v15.2 for Windows (MedCalc Software). The κ coefficient was calculated to determine the magnitude of the statistical agreement between two tests [25].

### Ethical statement

Blood sample collections were performed in accordance with the Brazilian Committee for animal care and use (COBEA) guidelines and approved by the Universidade Federal de Pelotas (UFPel) Ethics Committee for animal research and by the Ethics Commission of the Instituto de Pesquisas Veterinárias Desidério Finamor (IPVDF) under protocols numbers 002129/2010 and 02/2009, respectively. Vaccination procedures (S1 Table) were also performed under protocol 002129/2010.

## Results

### Development of rgD5ELISA and ROC analysis

**Fig 1:**
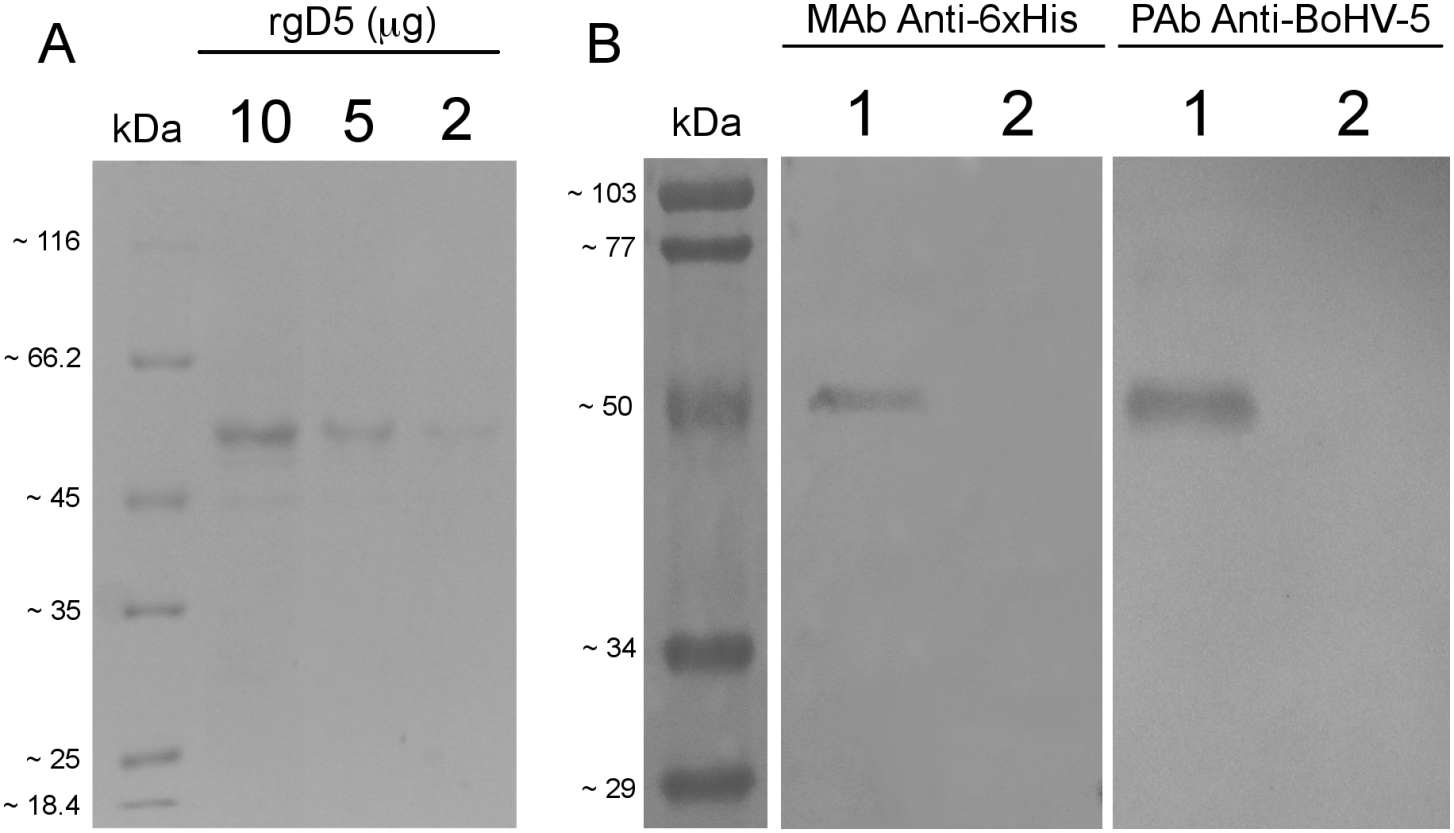
Coomassie Blue Staining and Western Blot Analysis of the Purified rgD5. (A) After purification steps, different concentrations of purified rgD5 protein (55 kDa) were separated by SDS-PAGE in a 12% gel and stained overnight with Coomassie Blue R-250. Lane kDa: Unstained Protein MW Marker (Fermentas/Thermo Fisher Scientific); Lane 10: 10 μg rgD5; Lane 5: 5 μg rgD5; Lane 2: 2 μg of rgD5; (B) Western blot was performed with mouse monoclonal antibody (MAb) Anti-6xHis HRP conjugated (Anti-6xHis) or with polyclonal antibodies (PAb) from bovine immunized with inactivated BoHV-5 (Anti-BoHV-5). Lane M: Prestained Low Range Protein MW Standard (Bio-Rad); Lane 1: Purified rgD5; Lane 2: KM71H supernatant of non-transformed yeast cells after methanol induction used as negative control.

**Fig 2:**
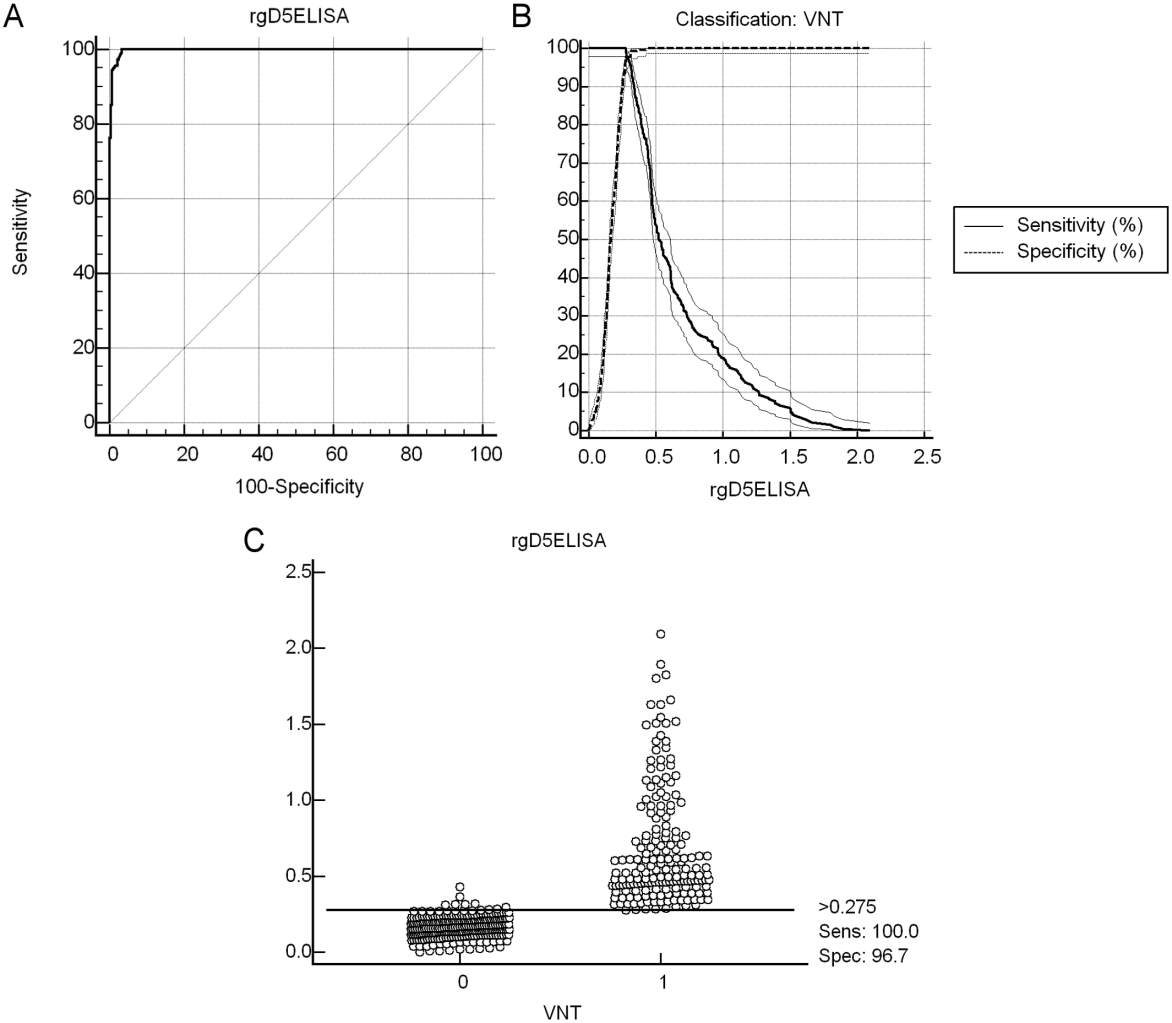
Receiver-Operating Characteristics Analysis of the Developed rgD5ELISA. (A) Receiver-operating characteristics (ROC) curves based on test results obtained for the rgD5ELISA (*n* = 450). (B) Relationship between ROC-based estimates for test sensitivity and specificity and rgD5ELISA cut-offs; (C) Interactive dot diagram based on rgD5ELISA outcomes in relation to virus neutralization test (VNT negative = 0 and VNT positive = 1).

The purification of the rgD5 and its antigenicity were respectively confirmed by Coomassie Blue protein staining (Fig 1A) and Western blot (Fig 1B) through visualization of the expected ~ 55 kDa protein band. The amount of the purified protein for plate coating was established at 50 ng/well and at this concentration, positive and negative sera were better differentiated into reagents (positive) or non-reagents (negative) when diluted 1:400. Bovine sera samples (*n* = 450) were tested by VNT, which revealed that 269 samples (59.8%) were found to be negative (CPE observed in the dilution 1:2) and 181 samples (40.2%) were positive to BoHV-5 (absence of CPE). Comparing to the results of the VNT, the AUC indicated that the rgD5ELISA test developed was on average 99.8% accurate (Fig 2A). The 95% confidence intervals (CI) of the AUC for the ELISA ranged from 98.7 to 100% and the significance level (Area = 0.5) was *P* < 0.0001. The ROC analysis showed that the optimal cut-off point was the O.D. 492 nm reading of 0.275, corresponding to a sensitivity of 100% and specificity of 96.7% (Fig 2B). Positive predicted values (PPV) and negative predictive values (NPV) were, respectively, 98.91% (95% CI 97.15–99.72) and 100% (95% CI 96.67–100), in a population with disease prevalence of approximately 75% [10]. Of the 181 VNT positive sera, all samples were considered to be true positive as the ELISA readings were above the optimized cut-off (Table 1). Among the 269 VNT negative sera, 10 samples (3.72%) were considered false positive since the ELISA readings showed values above the cut-off; 259 samples (96.28%) showed ELISA readings below the cut-off and were considered to be true negative (Fig 2C). A κ coefficient of 0.954 (95% CI 0.926–0.982) was calculated between VNT and rgD5 ELISA.

**Table 1:** Comparison Between the Virus Neutralization Test and the rgD5ELISA for the Detection of Antibodies to BoHV-5.

	rgD5 ELISA		
VNT	Negative	Positive	Total
**Negative**	259	10	269 (59.8%)
**Positive**	0	181	181 (40.2%)
**Total**	259 (57.6%)	191 (42.4%)	450

The table presents the results of 450 sera analyzed by VNT and rgD5ELISA.

Kappa coefficient = 0.954 (95% CI 0.926–0.982).

## Discussion

In this study, the application of the recombinant BoHV-5 gD (rgD5) as antigen in the development of an indirect ELISA is described. The rgD5 expression in *P*. *pastoris* has been previously reported, showing that the truncated recombinant protein possess 312 amino acids (aa 43 to 354) from native BoHV-5a gD and one N-glycosylation site [21]. Antigenic and immunogenic characteristics of the rgD5 were confirmed by the stimulation of neutralizing antibodies in mice [26] and bovine (I. L. Araujo et al., unpublished data) vaccinated with an experimental subunit vaccine using the rgD5 as antigen. During infection, the gD interacts with host-cell receptors, allowing fusion of the viral envelope with the permissive cell membranes. As gD participates in the initial steps of infection and is a major target of host immune system, its use as antigen in vaccine development has been widely documented (reviewed in reference [20]).

To test the hypothesis that the rgD5 may be suitable for use as antigen in an immunodiagnostic test, a total of 450 serum samples were screened by the developed rgD5ELISA and by VNT, which is considered the “gold standard” diagnostic method for detection of antibodies to BoHV-1 and BoHV-5. The rgD5ELISA revealed excellent level of agreement with the VNT (Table 1), 96.7% specificity, 98.91% PPV, 100% sensitivity and 100% NPV. The developed rgD5ELISA uses a single dilution of the serum tested, offering considerable advantage over more labor-intensive assays, such as VNT.

The development of ELISAs using recombinant protein expressed in *P*. *pastoris* have been reported in several studies in the past decades, including assays for pseudorabies [27], herpes simplex virus 1 (HSV-1) [28], classical swine fever virus [29], reticuloendotheliosis virus [30] and influenza A virus subtype H5N2 [31]. The development of indirect and gB blocking ELISAs (gB-ELISAs) aiming the detection of antibodies against BoHV-1 has also been widely documented [32–34]. A comparative study of tests for diagnosis of BoHV-1 [35], revealed a low performance of most of the indirect ELISAs, when compared to both VNT and gB-ELISAs. Overall, those indirect tests presented 87% sensitivity and 99% specificity and most scored negatively samples originated from vaccinated animals with low antibody response, as did the VNT [35]. In contrast, 87.5% of samples from eight animals (*n* = 8) undergoing a BoHV-5 vaccination study collected 13 days post-vaccination (d.p.v) were scored as positive by the rgD5ELISA, while VNT did not detected neutralizing antibodies in these samples, which were expected to have low antibody response (S2 Table). The 96.7% specificity obtained by the rgD5ELISA could be attributed to 10 false-positive readings obtained in the serum analyses, which can be a representation of weakly positive animals to BoHV-5 that are not detectable by VNT, but could be considered positive by the rgD5ELISA.

To the best of our knowledge, there is no commercial ELISA that employs BoHV-5 antigens. The wider distribution of BoHV-1 in relation to BoHV-5 may offer an explanation for the absence of BoHV-5 antigens in commercially available immunodiagnostic tests, as BoHV-5 infections seems to be more prevalent in Australia and in South America [10]. The relatively recent attention drawn to BoHV-5 infections, the low prevalence of BoHV-5 in countries of the northern hemisphere and the high degree of antigenic cross-reactivity between these two virus types may also contribute to the absence of BoHV-5 specific assays.

In summary, the rgD5ELISA here proposed displayed an excellent correlation with VNT and was shown to be a simple, convenient and highly specific and sensitive virus-free assay, suitable for the detection of antibodies to BoHV-5 infections. However, the developed rgD5ELISA, due to the homology between the gD of BoHV-1 and BoHV-5 [36,37], cannot exclude cross-reactivity, as most of the available tests for BoHV-1 [35]. The rgD5ELISA may be of aid for the serological diagnosis of BoHV-5 infections, allowing large-scale sero-monitoring following vaccination and for sero-surveillance for BoHV-5 antibodies.

## Supporting Information

S1 Table Geographical Distribution of Brazilian Cattle Farms Where Sera Samples Were Collected. (PDF)

S2 Table Comparative Evaluation Between Virus Neutralization Test (VNT) and the rgD5ELISA for the Detection of Antibodies to BoHV-5 in Vaccinated Cattle (*n* = 8). (PDF)

## References

[1] Pidone CL, Galosi CM, Echeverria MG, Nosetto EO, Etcheverrigaray ME. Restriction endonuclease analysis of BHV-1 and BHV-5 strains isolated in Argentina. J Vet Med B Infect Dis Vet Public Health. 1999;46: 453–456. 10.1046/j.1439-0450.1999.00257.x 10528541

[2] D’Arce RCF, Almeida RS, Silva TC, Franco AC, Spilki F, Roehe PM, et al Restriction endonuclease and monoclonal antibody analysis of Brazilian isolates of bovine herpesvirus types 1 and 5. Vet Microbiol. 2002;88: 315–324. 12220807 10.1016/s0378-1135(02)00126-8

[3] Varela APM, Holz CL, Cibulski SP, Teixeira TF, Antunes DA, Franco AC, et al Neutralizing antibodies to bovine herpesvirus types 1 (BoHV-1) and 5 (BoHV-5) and its subtypes. Vet Microbiol. 2010;142: 254–260. 10.1016/j.vetmic.2009.10.016 19926411

[4] Jones C. Herpes simplex virus type 1 and bovine herpesvirus 1 latency. Clin Microbiol Rev. 2003;16: 79–95. 12525426 10.1128/CMR.16.1.79-95.2003 PMC145298

[5] Vogel FSF, Caron L, Flores EF, Weiblen R, Winkelmann ER, Mayer SV, et al Distribution of bovine herpesvirus type 5 DNA in the central nervous systems of latently, experimentally infected calves. J Clin Microbiol. 2003;41: 4512–4520. 14532175 10.1128/JCM.41.10.4512-4520.2003 PMC294956

[6] Studdert MJ. Bovine encephalitis herpesvirus. Vet Rec. 1989;125: 584. 2557703

[7] Salvador SC, Lemos RAA, Riet-Correa F, Roehe PM, Osório ALAR. Meningoencefalite em bovinos causada por herpesvírus bovino-5 no Mato Grosso do Sul e São Paulo. Pesqui Vet Bras. 1998;18: 75–82.

[8] Campos FS, Franco AC, Hübner SO, Oliveira MT, Silva AD, Esteves PA, et al High prevalence of co-infections with *bovine herpesvirus* 1 and 5 found in cattle in southern Brazil. Vet Microbiol. 2009;139: 67–73. 10.1016/j.vetmic.2009.05.015 19560292

[9] Claus MP, Alfieri AF, Alfieri AA. Herpesvírus bovino tipo 5 e meningoencefalite herpética bovina. Semina: Ciências Agrárias. 2002;23: 131–141.

[10] Claus MP, Alfieri AF, Médici KC, Lunardi M, Alfieri AA. Bovine herpesvirus 5 detection by virus isolation in cell culture and multiplex-PCR in central nervous system from cattle with neurological disease in Brazilian herds. Braz J Microbiol. 2007;38: 485–490.

[11] Delhon G, Moraes MP, Lu Z, Afonso CL, Flores EF, Weiblen R, et al Genome of bovine herpesvirus 5. J Virolol. 2003;77: 10339–10347. 10.1128/JVI.77.19.10339-10347.2003 PMC228503 12970418

[12] Esteves PA, Dellagostin OA, Pinto LS, Silva AD, Spilki FR, Ciacci-Zanella JR, et al Phylogenetic comparison of the carboxy-terminal region of glycoprotein C (gC) of bovine herpesviruses (BoHV) 1.1, 1.2 and 5 from South America (SA). Virus Res. 2008;131: 16–22. 17889957 10.1016/j.virusres.2007.08.004

[13] Traesel CK, Sá e Silva M, Weiss M, Spilki FR, Weiblen R, Flores EF. Genetic diversity of 3’ region of glycoprotein D gene of bovine herpesvirus 1 and 5. Virus Genes. 2014;48: 438–447. 10.1007/s11262-014-1040-5 24482291

[14] Vogel FS, Flores EF, Weiblen R, Kunrath CF. Atividade neutralizante anti-herpesvírus bovino tipos 1 (BHV-1) e 5 (BHV-5) no soro de bovinos imunizados com vacinas contra o BHV-1. Cienc Rural. 2002;32: 881–883.

[15] Del Médico Zajac MP, Puntel M, Zamorano PI, Sadir AM, Romera SA. BHV-1 vaccine induces cross-protection against BHV-5 disease in cattle. Res Vet Sci. 2006;81: 327–334. 16540133 10.1016/j.rvsc.2006.01.004

[16] House JA, Baker JA. Bovine herpesvirus IBR-IPV. The antibody virus neutralization reaction. Cornell Vet. 1971;61: 320–335. 4325253

[17] Takiuchi E, Alfieri AF, Alfieri AA. Herpesvírus bovino tipo 1: Tópicos sobre a infecção e métodos de diagnóstico. Semin-Cienc Agrar. 2001;22: 203–209.

[18] Collins JK, Bulla GA, Riegel CA, Butcher AC. A single dilution enzyme-linked immunosorbent assay for the quantitative detection of antibodies to bovine herpesvirus type 1. Vet Microbiol. 1985;10: 133–147. 2984835 10.1016/0378-1135(85)90015-x

[19] Balamurugan V, Venkatesan G, Sen A, Annamalai L, Bhanuprakash V, Singh RK. Recombinant protein-based viral disease diagnostics in veterinary medicine. Expert Rev Mol Diagn. 2010;10: 731–753. 10.1586/erm.10.61 20843198

[20] Alves Dummer L, Pereira Leivas Leite F, van Drunen Littel-van den Hurk S. Bovine herpesvirus glycoprotein D: A review of its structural characteristics and applications in vaccinology. Vet Res. 2014;45: 111 10.1186/s13567-014-0111-x 25359626 PMC4252008

[21] Babiuk LA, van Drunen Littel-van den Hurk S, Tikoo SK. Immunology of bovine herpesvirus 1 infection. Vet Microbiol. 1996;53: 31–42. 9010996 10.1016/s0378-1135(96)01232-1

[22] Dummer LA, Conceição FR, Nizoli LQ, Moraes CM, Rocha AR, Souza LL, et al Cloning and expression of a truncated form of envelope glycoprotein D of bovine herpesvirus type 5 in methylotrophic yeast *Pichia pastoris*. J Virol Methods. 2009;161: 84–90. 10.1016/j.jviromet.2009.05.022 19501621

[23] Souza VF, Melo SV, Esteves PA, Schmidt CS, Gonçalves DA, Schaefer R, et al Caracterização de herpesvírus bovinos tipos 1 (BHV-1) e 5 (BHV-5) com anticorpos monoclonais. Pesqui Vet Bras. 2002;22: 13–18.

[24] Fischer G, Cleff MB, Dummer LA, Paulino N, Paulino AS, Vilela CO, et al Adjuvant effect of green propolis on humoral immune response of bovines immunized with bovine herpesvirus type 5. Vet Immunol Immunopathol. 2007;116: 79–84. 17275918 10.1016/j.vetimm.2007.01.003

[25] Alvarado JF, Dolz G, Herrero MV, McCluskey B, Salman M. Comparison of the serum neutralization test and a competitive enzyme-linked immunosorbent assay for the detection of antibodies to vesicular stomatitis virus New Jersey and vesicular stomatitis virus Indiana. J Vet Diagn Invest. 2002;14: 240–242. 12033681 10.1177/104063870201400309

[26] Dummer LA, Araujo IL, Finger PF, Santos AG Jr, da Rosa MC, Conceição FR, et al Immune responses of mice against recombinant bovine herpesvirus 5 glycoprotein D. Vaccine. 2014;32: 2413–2419. 10.1016/j.vaccine.2014.03.011 24657716

[27] Ao J, Wang J, Chen X, Wang X, Long Q. Expression of pseudorabies virus gE epitopes in *Pichia pastoris* and its utilization in an indirect PRV gE-ELISA. J Virol Methods. 2003;114: 145–150. 14625049 10.1016/j.jviromet.2003.09.012

[28] Wang ZY, Wen HL, Tao ZX. ELISA study on application of recombinant glycoprotein D of herpes simplex virus-1 in diagnosis of herpes simplex virus-1 infection with ELISA. Zhonghua Shi Yan He Lin Chuang Bing Dy Xue Za Zhi. 2005;19: 159–161. 16027786

[29] Huang C, Chien M-S, Hu C-M, Chen C-W, Hsieh P-C. Secreted expression of the classical swine fever virus glycoprotein E(rns) in yeast and application to a sandwich blocking ELISA. J Virol Methods. 2006;132: 40–47. 16213600 10.1016/j.jviromet.2005.08.020

[30] Li K, Gao H, Gao L, Qi X, Gao Y, Qin L., et al Development of an indirect ELISA for serological detection of reticuloendotheliosis virus using the gp90 protein expressed in *Pichia pastoris*. J Virol Methods. 2012;180: 43–48. 10.1016/j.jviromet.2011.12.008 22207082

[31] Shehata AA, Fiebi P, Sultan H, Hafez M, Liebert UG. 2012 Development of a recombinant ELISA using yeast (Pichia pastoris)-expressed polypeptides for detection of antibodies against avian influenza A subtype H5. J Virol Methods. 2012;180: 18–25. 10.1016/j.jviromet.2011.12.004 22197190

[32] Bashir S, Singh R, Sharma B, Yadav SK. Development of a sandwich ELISA for the detection of bovine herpesvirus type 1. Asian Pac J Trop Med. 2011;4: 363–366. 10.1016/S1995-7645(11)60104-1 21771677

[33] Oliveira SAM, Brum MCS, Anziliero D, Dellagostin O, Weiblen R, Flores EF. Prokaryotic expression of a truncated form of bovine herpesvirus 1 glycoprotein E (gE) and its use in an ELISA for gE antibodies. Pesqui Vet Bras. 2013;33: 41–46.

[34] Kramps JA, Magdalena J, Quak J, Weerdmeester K, Kaashoek MJ, Maris-Veldhuis MA, et al A simple, specific, and highly sensitive blocking enzyme-linked immunosorbent assay for detection of antibodies to bovine herpesvirus 1. J Clin Microbiol. 1994;32: 2175–2181. 7529249 10.1128/jcm.32.9.2175-2181.1994 PMC263962

[35] Kramps JA, Banks M, Beer M, Kerkhofs P, Perrin M, Wellenberg GJ, et al Evaluation of tests for antibodies against bovine herpesvirus 1 performed in national reference laboratories in Europe. Vet Microbiol. 2004;102: 169–181. 15327792 10.1016/j.vetmic.2004.07.003

[36] Abdelmagid O, Minocha H, Collins J, Chowdhury S. Fine mapping of bovine herpesvirus-1 (BHV-1) glycoprotein D (gD) neutralizing epitopes by type-specific monoclonal antibodies and sequence comparison with BHV-5 gD. Virology. 1995;206: 242–253. 7530392 10.1016/s0042-6822(95)80039-5

[37] Gabev E, Tobler K, Abril C, Hilbe M, Senn C, Franchini M, et al Glycoprotein D of bovine herpesvirus 5 (BoHV-5) confers an extended host range to BoHV-1 but does not contribute to invasion of the brain. J Virol. 2010;84: 5583–5593. 10.1128/JVI.00228-10 20219909 PMC2876591

