# Supplementary material for: Development of an Indirect ELISA for Serological Diagnosis of Bovine herpesvirus 5
Source: PLoS One. 2016 Feb 11;11(2):e0149134. doi: 10.1371/journal.pone.0149134 (PMC4750905; doi:10.1371/journal.pone.0149134)
Supplement: S1 Table — (PDF) [file pone.0149134.s001.pdf]

S1 Table. Geographical Distribution of Brazilian Cattle Farms Where Sera Samples Were Collected.

Geographical Coordinates	City	Number of samples
30º52’06.6”S, 51º50’39.1”W	Camaquã	15
31º51’39.0”S, 52º25’35.5”W	Capão do Leão	17
31º48’01.4”S, 52º39’33.8”W	Capão do Leão	6
30º59’07.3”S, 54º38’19.6”W	Dom Pedrito	26
30º50’21.0”S, 54º39’11.0”W	Dom Pedrito	120
30º03’05.3”S 51º18’58.0”W	Eldorado do Sul	73
31º55’34.8”S, 52º40’52.0”W	Pedro Osório	112
31º53’51.0”S, 52º54’58.1”W	Pedro Osório	56
31º55’16.6”S, 52º42’39.2”W	Pedro Osório	25
