# Supplementary material for: Development of an Indirect ELISA for Serological Diagnosis of Bovine herpesvirus 5
Source: PLoS One. 2016 Feb 11;11(2):e0149134. doi: 10.1371/journal.pone.0149134 (PMC4750905; doi:10.1371/journal.pone.0149134)
Supplement: S2 Table — (PDF) [file pone.0149134.s002.pdf]

S2 Table. Comparative Evaluation Between Virus Neutralization Test (VNT) and the rgD5ELISA for the Detection of Antibodies to BoHV-5 in Vaccinated Cattle (*n* = 8).

	Days post vaccination[a]						
	**0**	**13**	**27**	**56**	**111**	**160**	**363**
**rgD5ELISA[c]**	0/8[b]	7/8	8/8	8/8	8/8	8/8	8/8
**VNT[d]**	0/8	0/8	8/8	8/8	8/8	8/8	8/8

[a] Two years old calves (n=8) were vaccinated with an experimental inactivated BoHV-5 vaccine containing $10^{5.5}$ $CCID_{50}$/ml (previous to inactivation) emulsified in 50% v/v Montanide ISA 50 V2 (Seppic Adjuvants). The vaccine was administered (3ml/dose) intramuscularly (IM) on days 0 (1st dose), 13 (2nd dose) and 357 (3rd dose). Samples collected at the indicated days post vaccination were analyzed with both VNT and rgD5ELISA.

[b] Positively reacting samples/ total number of samples tested.

[c] Samples with O.D. (492 nm) above the cut-off point (0.275) were considered positive.

[d] Samples with VNT titers < 2 were considered negative
